# Evolution of Sensory Properties of Beef during Long Dry Ageing

**DOI:** 10.3390/foods11182822

**Published:** 2022-09-13

**Authors:** Ellies-Oury Marie-Pierre, Grossiord Benoit, Denayrolles Muriel, Papillon Sandrine, Sauvant Patrick, Hocquette Jean-François, Aussems Emmanuel

**Affiliations:** 1 INRAE, Clermont-Ferrand, VetAgro Sup, UMR1213, Recherches sur les Herbivores, 63122 Saint Genès Champanelle, France; 2 Bordeaux Sciences Agro, 1 Cours du Général de Gaulle, CS 40201, 33175 Gradignan, France; 3 UMR CNRS 5248, CBMN, Université de Bordeaux, 33160 Pessac, France; 4 JA Gastronomie, ZI de la Romanerie Rue du Paon, 49124 Saint Barthélemy d’Anjou, France

**Keywords:** tenderness, toughness, bovine, meat, colour, compression, shear force

## Abstract

Ageing is an essential step in obtaining meat with satisfactory sensory properties. Dry-ageing, although being a niche practice, is increasingly being developed to enhance the taste experience of meat consumers. In this work, we studied the kinetics of the evolution of muscle properties with increasing ageing time, in order to propose an optimal duration, allowing a compromise between quality and meat weight loss reduction. Our study was performed on 32 samples from 8 animals for which the *Longissimus thoracis* sensory properties were analysed at different stages of ageing (7, 16, 35 and 60-days post-slaughter). This work showed an increase in the dry matter content of meat with increasing ageing duration, concomitant with a slight increase in pH. Although the luminance of the meat is stabilized after 14-days, the red and yellow indices decrease until 35-days of ageing. Iron content also decreases with ageing duration. Finally, the kinetic evolution of muscle rheological properties indicates that the toughness decreases at least up to 35-days on raw meat. Cooking seems to homogenise the tenderness of the samples, no difference was noticed between the different ageing durations when meat was cooked. These first experimental data need to be confirmed with different animal types.

## 1. Introduction

Ageing is defined as all the changes that the meat undergoes during storage, after the rigor mortis has set in. During this period, beef muscles undergo several changes that are responsible for the development of the sensorial qualities of the meat [1]. Indeed, ageing allows to obtain an increase in tenderness, juiciness and flavour of beef [2,3,4]. The colour is also modified in link with myoglobin oxygenation and oxidation processes [5]. Meat colour can be influenced by many factors that are interrelated and can lead to important visual changes and ultimately, influence consumers’ perception of quality and freshness, such as temperature, packaging conditions, lipid oxidation during aging (Tomasevich et al., 2021). According to King et al. (2012), aging significantly impacts colour-life but animal variation of colour stability appears to be consistent across aging times: the colour stability of steaks sampled just after grading can provide that of aged steaks.

Among sensory descriptors, colour play a key role in the consumer’s buying act because it is an indicator of freshness [1]. pH has an important effect on pigments (e.g., chlorophyll, carotenoids, anthocyanins, etc.) responsible for meat colour [5,6].

Tenderness has been repeatedly reported as the most important sensory descriptor of beef, although flavour liking becomes more important in recent years [7]. Indeed, tenderness plays a key-role on sensory perception and consumer meat acceptance. Texture measurements are associated with the consumer’s meat perception after mastication and manipulation prior to ingestion. However, a number of studies have shown that a significant portion of retail meat can be considered tough. As a result, a large segment of consumers is willing to pay a premium for guaranteed tender meat [8]. Meat texture is generally difficult to evaluate using physico-chemical tests, as texture depends on the state and interactions of different muscle components, especially myofibrillar and connective tissues. Thus, apart from measuring shear force, there is no reliable method of predicting tenderness [9,10], except the Meat Standards Australia grading scheme which predicts a global eating score which is in fact a combination of tenderness, flavour liking, juiciness and overall liking [11].

Ageing period is mainly characterised by biochemical and physicochemical transformations such as degradation of contractile proteins, which results in changes in mechanical properties [12]. These modifications are mainly linked to the activity of proteolytic enzymes, which are more activated by pH lowering and which will allow protein degradation and hence improvement of tenderness [10,13]. The acidification of the muscle is the result of anaerobic glycolysis. Thus, the ultimate pH (pHu) is a crucial criterion for the proper development of ageing, and must be located between 5.5 and 5.8 [12].

A large number of studies have shown that the calpain proteolytic system (that consists of μ-calpain, m-calpain, and calpain-3; but also calpain inhibitor, the calpastatin) plays a key role in post-mortem proteolysis and tenderization. Cathepsins but also caspases are also important in ageing process. These enzymes act on the structure of myofibrils by degrading myofibrillar proteins namely desmin, skelemin and obscurin [12]. These enzymes have been shown to be the first enzymes to be involved in the degradation of structural proteins in link with the initial mechanisms of programmed cell death.

Ageing might be wet or dry, depending on the technology and processes employed [2,4]. Wet ageing is the most widely used ageing process due to its ease of implementation and cost, which remain reasonable and affordable for many stakeholders. This allows the sale price of meat from this process to be much more affordable than meat from long dry ageing [14]. For wet ageing, carcasses are cut up, the samples are vacuum packed and then stored under refrigerated conditions for up to 35 days [14].

The new techniques of long dry ageing of meat is an innovation that not only allows the improvement of sensory and taste qualities by offering consumers a product with a particular taste [14,15], but could also allow for an increase in the shelf life of the product [16]. Thus, in order to meet the new expectations of consumers, who have become increasingly demanding in terms of sensory quality, the meat industry has resorted to that new meat ageing process [17] dry ageing.

For dry ageing, beef carcasses or unpackaged pieces are directly stored under strictly controlled environmental conditions in terms of temperature, relative humidity and air flow [14]. The majority of research confirms that the control of environmental conditions (temperature, relative humidity and time) is crucial for successful dry ageing, and for obtaining meat with the desired organoleptic quality [2,4,18].

The application of dry ageing for a significant period of time, more than 21 days post-slaughter, results in meat with superior taste and better nutritional quality [19]. Usually meat consumers have previously indicated that they are willing to pay more for this type of meat and might also prefer the flavour of meat with long dry ageing to that of vacuum-matured meat (wet ageing), once they become familiar with this type of meat [14]. Nevertheless, the production of this type of meat, which is dry-aged for a long time, requires the implementation of a very heavy, complex and difficult to control process that requires the control of several factors (temperature, relative humidity and ventilation [4,20]. In addition, this ageing leads to a significant loss in weight and material, which reduces the production yield [4]. The conditions of ageing of the carcasses are, moreover, at the origin of a significant loss of weight of the meat, related with the desiccation of the carcasses. This production cost explains a high selling price of between €65 and €200 per kilo for some beef cuts (i.e., more than 2.5 to 8 times the price of a commonly marketed piece), with dry-aged beef costing around 25% more than wet-aged beef [21].

Researchers agreed on the optimal temperature range to be applied, which is between 0 and 4 °C, based on the fact that dry ageing temperatures should not differ from those of vacuum ageing [17,18,20,21,22]. A relative humidity of 61 to 85% is recommended with careful monitoring throughout the ageing process [14]. Indeed, any fluctuation could have an impact on the microbiological quality [22]. Despite the many studies that have been conducted, there has been no determination of the optimal duration to apply for ageing. The most common duration used for the production of dry-aged meat is between 14 and 40 days [17]. Some researchers have tested the impact of longer ageing times on the sensory quality of the meat. For example, [22] tested an ageing time of up to 120 days. They concluded that the optimal length of time was between 50 and 80 days in order to achieve the tenderness, taste and juiciness sought by consumers.

In order to optimise ageing costs and meat quality, the aim of this work carried out was to monitor, in kinetics, the evolution of the sensory properties of the meat according to dry-ageing duration in order to estimate more accurately an optimal duration for dry-ageing of beef.

## 2. Material and Methods

### 2.1. Material

To carry out this study, we worked with animals that were representative of those usually selected for long ageing by professionals in the sector, i.e., sufficiently fat animals (according to the expert view of the slaughterhouse professionals) with a good conformation. For this preliminary study and because of the constraints linked to the Coronavirus pandemic, we worked with 8 animals (being both Angus and Angus crossbred animals), together with 4 ageing times which means a total number of 32 samples. For each of them, the information available on the identification form was recorded (age, gender, weight, conformation, fatness and breed) (Table 1).

Animal carcasses were assessed after 24 h of post-mortem chilling in a refrigerated room with an average temperature between 0 and 4 °C. Then, they were assessed according to the EUROP grid (European conformation and fat scores were both converted into a continuous 15-point scale) [23]. Marbling was assessed at the 5th rib of the carcass [11,24] according to specifications of the ABCAS (Australian Beef Chiller Assessment System) Reference Standards, a benchmark for the measurement of the main quality characteristics of the bovine carcasses adopted by the UNECE Bovine Language standards. These chiller assessment standards are disseminated in Europe by the International Research Meat 3G Foundation (IMR3GF). The Meat Standards Australia (MSA) score ranges from 100 to 1190 in increments of 10 and indicates the amount, the size, the fineness, and the distribution of fat inclusions in muscles. The assessment was done by two 3G chiller assessors accredited by the IMR3GF. The rib eye was exposed to air for at least 20 min up to 3 h after cutting, allowing the meat to bloom before the MSA marbling assessment that was carried out using a visual standard.

At 48 h post-mortem, the 4-rib rack was cut from the right half carcass. It was then stored in the dry ageing room at a temperature of 0–2 °C, 5 m/s ventilated cooling and 80–87% relative humidity for 60 days. From this square, a 5 cm thick rib was taken at 7, 16, 35 and 60 days of ageing (respectively T1-T2-T3 and T4). The samples were taken under conditions as close as possible to “sterility”, in order to limit microbial contamination by the operator and the environment, unrelated to the ageing process. All analyses were carried out on fresh meat.

### 2.2. Methods

The dry matter at each ageing-time was assessed after 24 h in a ventilated oven at 104 °C. The dry matter content is given by the following relationship:

[(*m*_2_ − *m*_0_)/(*m*_1_ − *m*_0_)] × 100 = % dry matter

with *m*_0_: weight of the empty tray; *m*_1_: weight of the full tray before steaming; *m*_2_: weight of the full tray after steaming.

For ultimate-pH analysis, a pH meter FiveGo (Mettler) with a puncture electrode was used. The pH meter was standardized by a two-point method against standard buffers of pH 4.0 and pH 7.0. The ultimate pH was measured 48 h after slaughter. As the carcasses were cut up and the samples taken in a commercial slaughterhouse after 48 h of refrigeration, it was impossible to obtain the final pH at 24 h. We therefore assumed that the pH of the carcass, before cutting, did not change or changed only slightly between 24 and 48 h.

The colour of raw rib was characterised by L*, a* and b* trichromatic values [25]. The colour of meat was assessed at 12 points for each sample using the CIE Lab system. It was measured on the surface of meat samples using a hand-held tri-stimulus colorimeter (Minolta Chroma Meter CR-400, Minolta, Osaka, Japan) with an 8 mm diameter measuring area, the equipment was calibrated on the Hunterlab colour space system using a standard white plate (Minolta calibration plate, *Y* = 92.6, *x* = 0.3136, *y* = 0.3196). Colour was described as coordinates: lightness (L: 100 = white, 0 = black), redness (a* ± red–green) and yellowness (b* ± yellow–blue) of the CIELab scale. In order to monitor beef colour changes over ageing, total colour change (ΔE) was calculated.

This parameter can be expressed as:

$$\Delta E=\sqrt{{\left(L_{i}^{*}-L_{j}^{*}\right)}^{2}+{\left(a_{i}^{*}-a_{j}^{*}\right)}^{2}+{\left(b_{i}^{*}-b_{j}^{*}\right)}^{2}}$$

where $L_{i}^{*}$, $a_{i}^{*}$ and $b_{i}^{*}$ represented the individual readings after defined storage condition.

The heme iron content in the raw meat was evaluated according to the method of [26]. The calibration range was used to determine maximum concentrations of 75 μg. It was prepared on the basis of chlorohemin (0.0584 g/L, i.e., 5 mg/L haem iron) with a solvent mixture of distilled water, acetone and hydrochloric acid in the proportions 4.5 vol/20 vol/0.5 vol respectively. The solution was stored in the dark for 24 h. After 24 h, the contents of the tubes were quickly filtered and read with a spectrometer at 512 nm.

Sensory quality was approached by rheological values of shear force and compression force at 20 and 80%. Rheological analysis was carried out on raw muscle, and on cooked muscle. In order to provide results representative of the usual conditions of cooking and consumption of this type of meat, the samples were cooked on a grill (cooking at 300 °C for 1 min 45 s). Compression force at 20 and 80% was analysed using a Shimadzu EZ-SX texturometer, coupled with Trapezium X software, operating in the compression mode and using a 25 kg load cell [27]. For rheological tests of shear force, the equipment was fitted with a Warner-Bratzler knife. Samples of 1 cm^2^ (square cross-section), with muscle fibres parallel to the longitudinal axis of the sample were placed on the table, under the V blade, and were cut through as the blade moved down with a constant speed (10 mm min^−1^). Results were expressed in Newton’s (N). For these determinations six replicates per storage time and packaging films were recorded.

### 2.3. Statistical Analysis

Development of statistical methods for repeated measures data has been an active area of research in the past two decades because of advancements in computing hardware and software [28]. In the present experiment, a repeated-measures mixed model was fitted using the R packages lme4 [29] and lmerTest [30]. For the purposes of the model, ageing-length was considered a fixed effect. The animal was considered as a random effect.

$$y_{i}=X_{i}\beta +Z_{i}b_{i}+\varepsilon_{i}$$

with $X_{i}\beta =\beta_{0}+\beta_{x_{1}}x_{1}+\ldots +\beta_{x_{k}}x_{k}$; β: fixed effects; $Z_{i}b_{i}=b_{1}x_{1}+\ldots +b_{k}x_{k}$; b: random effects; $\varepsilon_{i}$: error.

This model used a random intercept to account for repeated measures of the same individual during ageing.

Two Principal Component Analysis (PCA) were then carried out according to [31] in order to characterize the links between muscular properties:

- whatever ageing duration (using a PCA with all the 4 × 8 = 32 samples): PCA1
- according to the evolutionary kinetics of the muscular properties with maturation (using a PCA with only 8 samples, the values for each muscular properties being equal to the difference calculated between T1 and T4): PCA2.

## 3. Results

### Results and Discussion

**Figure 1:**
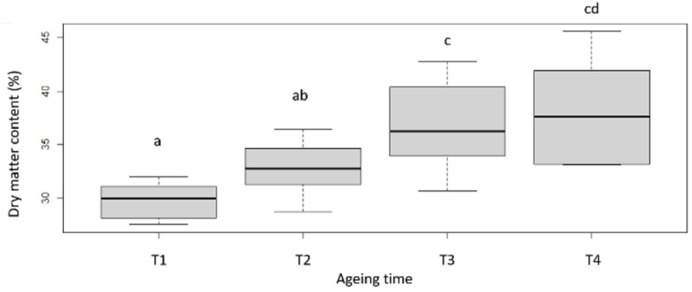
DM evolution with ageing time. T1, T2, T3 and T4 correspond with the different ageing length (respectively 7, 16, 35 and 60 days). Box-plot explanation: the central value represents the median; the edges of the rectangle are the quartiles and the extremities are calculated using 1.5 times the interquartile range. a, b, c, d: significant differences (*p* < 0.05).

**Figure 2:**
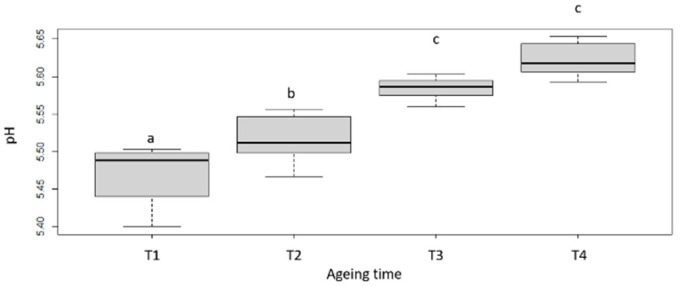
pH evolution with ageing time. T1, T2, T3 and T4 correspond with the different ageing length (respectively 7, 16, 35 and 60 days). Box-plot explanation: the central value represents the median; the edges of the rectangle are the quartiles and the extremities are calculated using 1.5 times the interquartile range. a, b, c: significant differences (*p* < 0.05).

Dry matter (DM) content of raw meat (Figure 1; Table 2), which was relatively homogeneous at T1 (ranking from 28 to 32%), tends to increase during ageing, in connection with the desiccation of the meat [4], but also to be more variable (with values ranking from 33 to 46%). The increase of raw meat dry matter is associated with a slight increase of the pH. Indeed, a slight increase in pH can be noted between T1 and T4 days of ageing as well as a homogenisation of the pH after 60 days of ageing around 5.62 ± 0.02 (Figure 2; Table 2). This evolution of the pH could possibly be produced by the nitrogenous components resulting from the proteolysis that takes place during ageing [32]. In the literature, the kinetics of pH evolution seem to be globally stable during ageing whether during the first 25 days, as previously noted by [33] also during the first 60 days of ageing as indicted by [17]. However, these conclusions are not in contradiction with our results, as the levels of variation between T1 and T4 remain quite limited (around 0.13 points).

**Figure 3:**
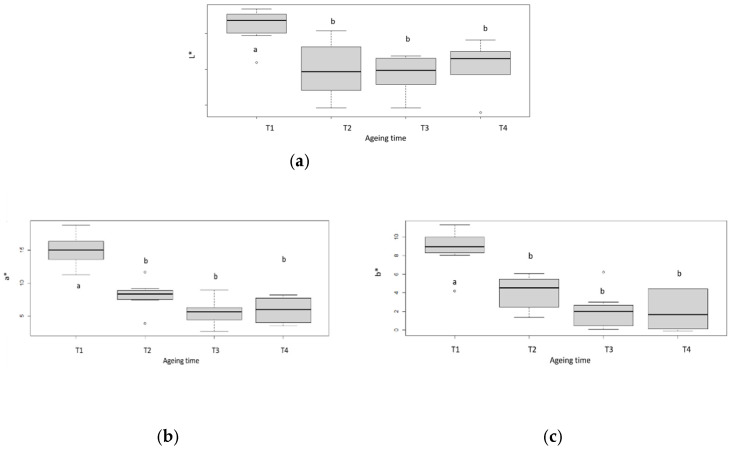
Box-plot of trichromatic coordinates (**a**): L*; (**b**): a*; (**c**): b* of rib muscles. T1, T2, T3 and T4 correspond with the different ageing length (respectively 7, 16, 35 and 60 days). Box-plot explanation: the central value represents the median; the edges of the rectangle are the quartiles and the extremities are calculated using 1.5 times the interquartile range. a, b: significant differences (*p* < 0.05).

The instrumental measurement of colour makes it possible to determine the luminance (L*), the hue and saturation from green to red (a*) and the hue and saturation from blue to yellow (b*) of a given sample (Figure 3). From the kinetic study of the evolution of the trichromatic colour coordinates, we observed a decrease in the values of a* and b* with ageing between 7 and 35 days (Figure 3b,c) and a stabilisation of the values of L* from 14 days of ageing (Figure 3a). This evolution favours the evolution of the colour towards a less red, less shiny and darker (more blackish) meat with ageing, confirming the results previously established. Indeed, it has been shown in several studies and through the instrumental measurement of colour that long dry ageing results in darker and less shiny meat and that this process contributes to the stabilisation of the colour when increasing the ageing time [34,35,36]. When calculating the colour differences ΔE between successive measurements (T1 and T2; T2 and T3; T3 and T4), it was found that the colour differences between different ageing times were always greater than 3 (data not shown). According to [37], when ΔE  >  3, colour differences are obvious for the human eye. Our results then indicate that the colour differences measured by the chromameter are perceptible to the human eye.

**Figure 4:**
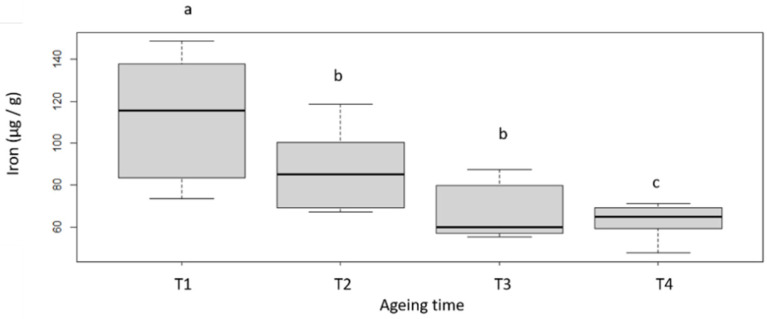
Iron content of the different samples as a function of ageing time. T1, T2, T3 and T4 correspond with the different ageing length (respectively 7, 16, 35 and 60 days). Box-plot explanation: the central value represents the median, the edges of the rectangle are the quartiles and the extremities are calculated using 1.5 times the interquartile range. a, b, c: significant differences (*p* < 0.05).

The heme iron content of our samples shows a great variability after 7 days of ageing, almost doubling (between 67.4 and 148.5 µg of iron/g of meat; Figure 4). It can be noted that lower values are found for females compared to males (data not shown). This result does not seem very logical, as beef from females is known to be brighter red than from males. However, it is also accepted that the amount of myoglobin varies between species and breeds, but also that it is dependent on animal age and sex and also on the type of muscle studied. As the number of animals is rather low, this aspect should be validated on a larger scale. Despite a high variability after 7 days of ageing, the profile of the evolution of the iron content is similar between the samples with a decrease observed for all of them, and a final content that is less variable after 60 days of ageing (T4: 76.5 ± 18.8 µg/g). Although [38] suggested that heme iron content is directly correlated with meat colour (i.e., meat is lighter with higher red intensity and lower yellow intensity when heme iron content is high), we were unable to establish such relationships between iron content and meat colour across different ageing times.

In the present experiment, tenderness kinetics during ageing was evaluated thanks to the shear force and the compression measurements (at 20 and 80% of the sample compression).

Shear force is an expression of the basic hardness of the meat, which cannot be reduced by extending ageing time since the connective tissue is not significantly affected by post-mortem changes [39]. Measurement of compression force gives an indication of the degree of ageing of the cut as this test can measure the respective contribution of the myofibres and the connective tissue to the tenderness of the meat [40].

Evolution of the raw meat samples showed a large variability in the shear and compression forces (both at 20 and 80%). This variability may be related to the fact that our samples are both small in size (in relation with the size of Angus and crossed-Angus animals) and heterogeneous (as they consist of both heifers and steers of various breeds).

**Figure 5:**
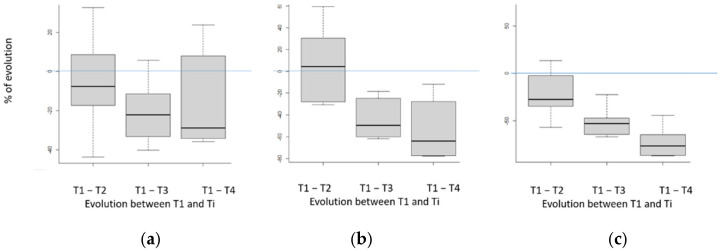
Kinetic of evolution for (**a**) shear force, (**b**) 20% compression shear, (**c**) 80% compression shear) depending on ageing time for raw meat expressed as a relative value of the content on day 7. T1, T2, T3 and T4 correspond to the different ageing times (respectively 7, 16, 35 and 60 days). Box-plot explanation: the central value represents the median; the edges of the rectangle are the quartiles and the extremities are calculated using 1.5 times the interquartile range.

**Figure 6:**
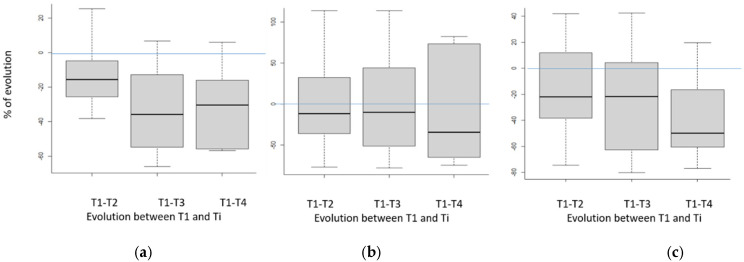
Kinetic of evolution for (**a**) shear force, (**b**) 20% compression shear, (**c**) 80% compressive shear) depending on ageing time for cooked meat expressed as a relative value of the content on day 7. T1, T2, T3 and T4 correspond to the different ageing times (respectively 7, 16, 35 and 60 days). Box-plot explanation: the central value represents the median; the edges of the rectangle are the quartiles and the extremities are calculated using 1.5 times the interquartile range.

Indeed, it is well known that steers and heifers from early-maturing breeds (such as Angus breed in particular) deposit significantly more intramuscular lipids than animals of continental bovine breeds. Thus, due to a negative genetic correlation between muscle development and tissue adiposity [41], these animals develop smaller muscle areas, and therefore smaller beef cuts at similar live weight [42]. Moreover, it has previously been established that tenderness and/or shear strength varied according to the age of the animals [43] and slightly according to the breed [44]. Due to this variability in absolute values, we expressed results as percentages of initial values (obtained after 7 days of ageing for the same animal) to highlight the decrease in strength with ageing time (Figure 5 and Figure 6).

According to our results, it is possible to demonstrate a reduction in the mean values of shearing and compressive force (at 20 and 80%) on raw meat with the increase in the ageing time (Figure 5). This reduction in the force necessary for shearing/compressing the meat reflects an improvement in the tenderness of the meat during ageing, as previously established in particular by [12,17,45,46]. This decrease in strength seems higher for the first days of ageing (up to 14 days) than beyond this period. The superposition of the boxplots obtained for shear force and 20% compression shear for the last two periods suggests that an increase in the meat ageing beyond 35 days would not lead to a significant decrease in hardness, as previously established by [21,47]. However, according to the results obtained on 80% compression shear, these conclusions must be confirmed on a larger number of individuals.

It is also interesting to observe that when the same forces are evaluated on cooked meat (Figure 6), all the box-plots differ to a smaller extent than on raw beef, suggesting that cooking has induced a homogenization of the hardness/tenderness of the samples. This questions homogeneity of cooking between the samples (despite standardized conditions) but also on potential effects of intramuscular lipid contents on shear force kinetics. It is all the more disturbing that, even if we know the importance of cooking on final beef tenderness, it is worrying that cooking is likely to negate the significant increase in tenderness of a sample thanks to long ageing, due to potential wrong cooking! In the future, it will be important to check these results on a larger population of carcasses in order to find an explanation for this phenomenon.

**Figure 7:**
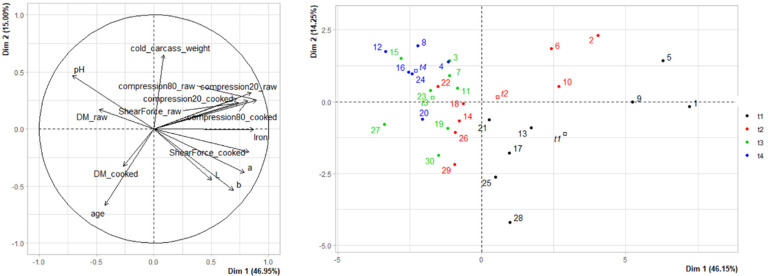
Correlation circle and projection of individuals for principal component analysis 1 performed on 4 × 8 = 32 samples. DM_raw, DM_cooked: Dry Matter on raw or cooked meat; ShearForce_raw, ShearForce_cooked: Shear Force measurements on raw or cooked meat; Compression20, Compression80: Compression shear at 20% or 80% of the sample thickness; L, a, b: trichromatic colour coordinates L*, a*, b*.

**Figure 8:**
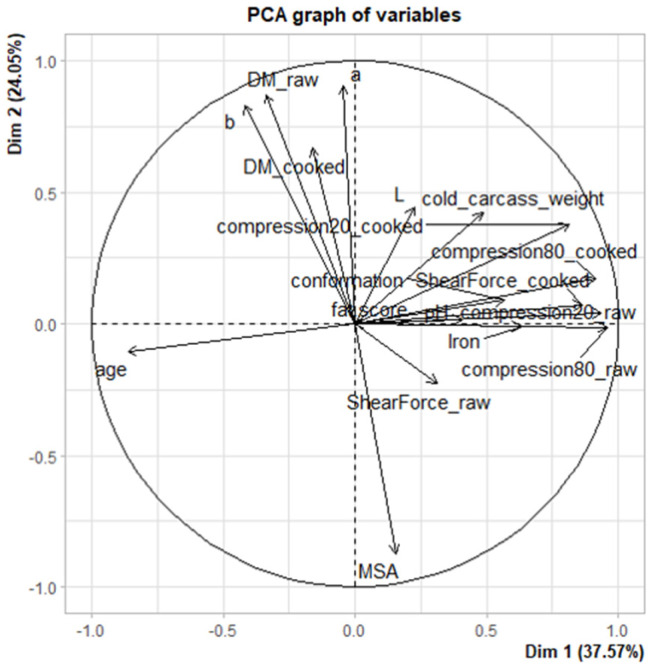
Correlation circle for principal component analysis 2 performed on 8 samples by using the difference between T1 and T4. DM_raw, DM_cooked: Dry Matter on raw or cooked meat; ShearForce_raw, ShearForce_cooked: Shear Force measurements on raw or cooked meat; Compression20, Compression80: Compression shear at 20 or 80% of the sample thickness; L, a, b: trichromatic colour coordinates L*, a*, b*. MSA: MSA marbling score.

Principal component analysis of the 4 × 8 = 32 samples (PCA1, Figure 7) shows correlations between rheology (shear and compression forces at 20 and 80%, on cooked and raw meat) and colour (L*, a* and b*) variables. The projection of individuals on axes 1–2 (representing 60.4% of the explained variance) reveals a heterogeneity of muscle properties at time T1, which is reduced with maturation. It thus seems that increasing the duration of maturation leads to a decrease in the variability of muscle properties, probably associated with a reduction in the heterogeneity of the sensory properties of the meat. This PCA also confirms the increase in pH and dry matter content with the maturation time. In parallel, with the increase in ripening time, a reduction and homogenisation of iron content, of the trichromatic values of colour L*, a* and b* and of the rheological values (compression and shear force) can be noted. The second PCA, carried out by calculating the difference in values between T1 and T4 for the different muscular properties (PCA2, Figure 8), shows that when age increases, the differences between the rheological values obtained between T1 and T4 decrease, implying that older animals value the increase in maturation time less than younger animals. However, this result needs to be confirmed, as the animals studied in this study were all less than 30 months of age (and therefore relatively young). In parallel, it can be noted that the increase in the marbling score assessed according to the MSA grading system is associated with a reduction in the differences in terms of dry matter and a* and b* coordinates. The most marbled animals have a more stable colour (in terms of yellow and red indices) with increasing maturation time.

To summarize, it is possible to describe the benefits and disadvantages of the long dry-ageing, stating the conclusions established in the literature (scientific and professional) as well as those from the present study. The main objective of dry-ageing is to produce a tender, full-flavoured beef that has the true ‘taste of beef’. Indeed, with this process, shear force and compression shear decrease (especially on raw meat), leading to a tenderness gain; juiciness increases; the pH and the dry matter increase; the flavours are more concentrated and the taste is more pronounced (nutty, rancid flavour). That’s why the lovers of mature dry beef are willing to pay more for it. However, this process also induces a shrinkage of the meat (up to 1/3 of its weight), an increase of production and purchase costs, an increase of microbial contamination risks and a loss of meat when cutting the crust (dry protective surface that acts as a barrier to microorganisms). Finally, this process induces technological constraints as it requires strict storage conditions for meat (temperature, pH, humidity, ventilation).

## 4. Conclusions

The objective of this work was to provide initial information on the kinetic evolution of the properties of meat with the practice of long-term dry ageing. The extension of the ageing time between 7 and 60 days leads to an evolution of the colour of the meat visible to the naked eye, which becomes less and less red, less shiny and darker. On the other hand, the evolution of the texture of the raw meat seems to be marked only during the first 35 days of ageing, therefore suggesting that a 35 days aging remains a good alternative. On the other hand, we also found that cooking seems likely to reduce the beneficial effects of this longer ageing time.

## Figures and Tables

**Table 1:** Animals characteristics at slaughter.

Animal	Type of Animal	Breed	Age (Months)	Cold Carcass Weight (kg)	Conformation (1–15 Scale)	Fat Score (1–15 Scale)	MSA Marbling Score
A	Steer	Angus × Charolais	24	443	U − (10)	3 = (8)	365
B	Steer	Angus	25	456	U = (11)	3 = (8)	475
C	Steer	Angus	24	415	U − (10)	3 = (8)	530
D	Heifer	Angus × Charolais	25	443	U = (11)	3 = (8)	358
E	Steer	Angus × Charolais	26	425	U − (10)	3 = (8)	543
F	Heifer	Angus × Charolais	26	457	U − (10)	3 = (8)	330
G	Heifer	Angus × Charolais	27	417	U − (10)	3 = (8)	485
H	Heifer	Angus × Charolais	27	400	R + (9)	3 = (8)	410

**Table 2:** DM and pH values for each ageing duration.

	T1	T2	T3	T4
DM (%)	29.8 ± 1.7 a	32.8 ± 2.5 ab	36.8 ± 4.2 c	38.2 ± 4.9 cd
pH	5.47 ± 0.04 a	5.52 ± 0.03 b	5.58 ± 0.01 c	5.62 ± 0.02 c

T1, T2, T3 and T4 correspond with the different ageing length (respectively 7, 6, 35 and 60 days). a, b, c, d: significant differences (*p* < 0.05).

## Data Availability

All the data are in the text.
